# Comparative Evaluation of Biofilm Formation on Temporization Crown Materials Used in the Rehabilitation of Primary Dentition With Different Polishing Materials: An In Vitro Study

**DOI:** 10.7759/cureus.68944

**Published:** 2024-09-08

**Authors:** Akansha Kaintura, Kavitha Ramar

**Affiliations:** 1 Pediatric and Preventive Dentistry, SRM (Sri Ramaswamy Memorial) Kattankulathur Dental College, Chennai, IND; 2 Pedodontics and Preventive Dentistry, SRM (Sri Ramaswamy Memorial) Kattankulathur Dental College, Chennai, IND

**Keywords:** biofilm formation, bis-acryl composite, bis-acryl resin, dental esthetics, dental restoration, finishing and polishing systems, streptococcus mutans adhesion, surface roughness

## Abstract

Introduction

Advancements in dental materials have enhanced aesthetic treatments for managing dental caries and injuries in primary dentition. Bis-acryl composite-based temporization materials are now preferred for restoring primary crowns due to their superior properties. However, prolonged exposure to dietary and hygienic factors can lead to discoloration and roughness, making efficient polishing essential to prevent plaque buildup.

Objective

This study aims to evaluate *Streptococcus mutans* biofilm formation on temporization material polished with different polishing systems.

Methods

This study tested bis-acryl methacrylate temporization material. Thirty disk-shaped specimens were prepared and divided into three groups according to the polishing system used (n = 10 per group): Shofu Super Snap mini kit (Shofu, San Marcos, CA), aluminum oxide polishing paste, and propol polishing paste. Each group’s specimens were polished according to the manufacturer’s instructions. Surface roughness (SR), scanning electron microscopy (SEM) morphological analysis, and *Streptococcus mutans* biofilm formation were assessed for each group.

Results

The results showed significant differences in roughness average (Ra) values among the polishing materials, with the Shofu Super Snap mini kit having the highest roughness (Ra = 2.04), followed by propol polishing paste (Ra = 1.30) and aluminum oxide paste (Ra = 0.75). Additionally, polishing methods significantly affected mean colony-forming unit (CFU) levels, with the first group having the highest mean CFU value (0.24), with SEM images showing substantial biofilm formation by *Streptococcus mutans*.

Conclusion

Bacterial biofilm formation on the aluminum oxide paste group’s surface differed from that on the propol polishing paste and aluminum oxide disc groups. The polishing techniques that we tested significantly influenced surface properties and biofilm formation. These findings suggest that selecting an appropriate polishing system can reduce the risk of gingival inflammation associated with temporization materials.

## Introduction

Advancements in dental materials have led to the development of numerous aesthetic treatment strategies for managing dental caries and traumatic injuries in primary dentition [1]. For restoring primary teeth, various therapeutic modalities have been proposed, including intra-coronal tooth-colored restorations such as glass ionomer cement, resin-modified glass ionomers (RMGIs), polyacid-modified resins, and resin composites. Full-coronal aesthetic restorations are also available, such as newly developed pre-fabricated primary zirconia crowns, celluloid strip crowns, and pre-veneered stainless steel crowns. Additionally, free-hand crown structure building using tooth-colored materials like RMGIs and composites is an option [2].

Bis-acryl resin, a temporization material, was introduced as an alternative for the fabrication and relining of temporary prosthetic devices. The advantages of this material include low exotherm values, reduced setting time, ease of handling, and convenience (as it can be administered directly in the mouth with an automix syringe) [3]. Recent advancements in the mechanical properties of bis-acryl composite-based temporization materials have made them the preferred choice for temporization applications. While these materials are commonly used for temporary crowns in permanent dentition, their application in primary dentition has also been explored. Given the short lifespan of primary teeth, bis-acryl composites offer a durable and cost-effective restoration option [4].

Bis-acryl resin provides stability, protection, and functionality to the restored tooth, influencing both the esthetic and therapeutic outcomes of the treatment plan [3]. However, prolonged exposure to factors such as diet, oral hygiene, water sorption, and chemical reactivity can increase the likelihood of discoloration and surface roughness [5]. Longer service durations highlight the importance of plaque prevention and the need for efficient polishing of temporization restorations, as emphasized by Borchers et al. [6]. If plaque is not removed, macromolecular byproducts such as butyrate and propionate, known for their toxic, antigenic, and enzymatic properties, can penetrate the basement membrane and cause an inflammatory response [7]. Balthazar reported that errors in crimping, contouring, and polishing stainless steel crowns can lead to high plaque deposits, increasing the risk of gingivitis [8].

Finishing and polishing techniques are crucial for maintaining aesthetics and producing smooth, polished prostheses from both biological and esthetic perspectives [3]. Smooth surfaces are easier to clean because they do not retain food particles, germs, or epithelial cells, reducing the risk of plaque buildup and its harmful effects on periodontal tissues [3]. According to Tupinambá et al., the type of material and polishing technique used to fabricate temporary dental prostheses significantly impact the quality of the final restoration [3].

To complete restorations, it is essential to remove excess coarse material from the temporization surface, provide proper anatomical contours, and achieve surface smoothing simultaneously [3]. Polishing involves specialized equipment and techniques, including silicone tips, abrasive tips with varying particle sizes, and chemical applications to the material's surface [3]. Oral bacteria primarily thrive by adhering to hard surfaces, such as teeth, filling materials, dental implants, and prostheses, each with unique surface properties [8].

Biofilm deposition on these surfaces can lead to secondary caries, denture stomatitis, and gingivitis [9]. Streptococcus mutans plays a key role in caries formation, and biofilms are more resilient than planktonic microbes. Temporization restorations typically exhibit greater surface roughness and less marginal adaptation compared to definitive restorations, resulting in increased biofilm adhesion [9]. The roughness of these surfaces protects microbes from saliva and masticatory forces, enhancing their initial adhesion to temporary restorative materials [9]. Adequate polishing can mitigate both plaque accumulation and surface roughness.

The initial adherence and retention of oral bacteria are strongly influenced by the surface-free energy and roughness of these surfaces. Research suggests that higher surface roughness or free energy on supragingival surfaces accelerates bacterial colonization and plaque formation, thereby increasing the risk of periodontal diseases [8]. Therefore, maintaining smooth surfaces with low free energy is crucial to reducing plaque buildup and minimizing the risk of caries and periodontitis [10].

Hence, this study aimed to assess how different polishing materials affect the degree of biofilm formation on the surface of temporary restorative materials.

Null hypothesis

There will be no change in biofilm formation on temporization materials using different polishing materials.

Alternate hypothesis

There would be a change in biofilm formation on temporization material using different polishing materials.

## Materials and methods

Temporization restorative materials play an essential role when performing indirect restorative procedures. They are acrylic-based (methyl-methacrylates, ethyl-methacrylates) and composite-based resins (bis-acryl composites, light-cured composites) [11]. In our study, we are using composite-based bis-acryl methacrylate resin. In the current study, temporary restorative materials were employed to measure surface roughness, assess the adhesion of bacteria and biofilm development, and capture scanning electron microscopy (SEM) pictures of biofilm formation on prepared specimen surfaces.

Ethical approval for this study was obtained from the Institutional Ethics Committee of SRM Medical College Hospital and Research Centre, with approval number SRMIEC-ST0323-13-2, on 05/04/2023.

**Figure 1:**
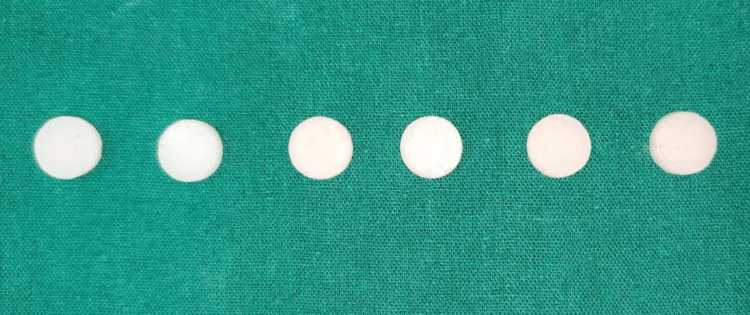
Prepared specimens.

The statistical software SPSS (version 27, IBM Corp., Armonk, NY) was used to determine the sample size targeting a 95% power and a 5% margin of error, which was 30 specimens. Figure 1 shows the disc-shaped specimens constructed by placing temporary restorative material into 1.5 mm thick and 6 mm diameter metal molds. Subsequently, Mylar strips were applied to both the upper and lower sides of the mold and the material was then pressed between two glass slides and cured for 20 seconds on all sides using a light-cure device (Woodpecker MINI S Curing Light, Guangxi, China) that was in direct contact with the Mylar strips.

Next, the specimens were randomly split into three groups based on the polishing methods used, and 10 specimens were allocated to each group.

All specimens were finished using diamond burs for 15 seconds each, as described below. The control group was polished with aluminum oxide polishing discs (Shofu Super Snap mini kit, Shofu, San Marcos, CA). Experimental groups 1 and 2 were polished with aluminum oxide paste (Prime Aster Compo, Prime, Thane, India) and propol polishing paste, respectively, using a prophy cup.

All specimens were then ultrasonically cleaned in distilled water for 10 minutes; they were first rinsed and then dried with oil-free air [12]. A single operator used a low-speed handpiece at 10,000 rpm for 15 seconds in one direction, with mild, consistent pressure, to accomplish all finishing and polishing processes. Every time a specimen was polished, a new piece of polishing material was used and subsequently discarded. Before the subsequent polishing or finishing procedure began, each specimen was cleansed with distilled water and allowed to air dry.

Surface roughness

After the surface treatments were finished, the average surface roughness of each specimen was determined using a stylus profilometer (Mitutoyo SJ 301, Mitutoyo, Kawasaki, Japan). The stylus of the instrument was moved around the surface of each specimen three times while applying constant pressure. The values of profile roughness average (Ra) for each specimen were computed and reported in μm. The profilometer was used at a resolution of 0.01 μm, while the transverse length was 5.5 mm, the interval (cut-off length) was 0.8 mm, and the speed of the stylus was 1 mm/s.

Microbiological procedures

Before being tested with bacteria, the disc specimens were individually packed, autoclaved for 15 minutes at 121°C, and cleaned.

Analysis by colony-forming units per milliliter (CFU/ml) counting

The specimens were incubated with the fresh broth suspension of *S. mutans*, and the biofilm was prepared for seven days by adding fresh broth on alternate days. The blocks were washed with sterile distilled water to remove planktonic cells after incubation, and swabs were taken from them. The swabs were made using lawn culture to sterile mutans sanguis agar, and the plates were incubated at 37°C for 24-48 hours. The colonies were counted and reported as colony-forming units per milliliter (CFU/ml) following incubation.

Analysis by SEM

After being fixed for one hour in 2.5% glutaraldehyde, two specimens from each group were placed in a 37°C bacteriological incubator to dry overnight. This made the biofilm visible on the surfaces of the specimens in SEM. The samples were given a platinum sputtering coating before they were closely examined at magnifications of 3000×, 5000×, and 6000× using JOEL FE-SEM IT800 (Tokyo, Japan), operating at 20 kV. A descriptive analysis was carried out after the recording of typical micrographs of the biofilm on the objects’ surface.

## Results

This in vitro study was conducted with the aim to evaluate *Streptococcus mutans* biofilm formation on temporization material polished with different polishing systems.

The data were analyzed using SPSS. Normality was assessed, and as the data followed a non-normal distribution, nonparametric tests were employed for the analysis. Mean and standard deviation were used to represent the average values for colony-forming units (CFU) and surface roughness. Inferential statistics were applied to compare the groups, with the Kruskal-Wallis test used to compare the mean values of the parameters. A p-value of 0.05 was set as the threshold for statistical significance.

Descriptive statistics

The overall mean for *Streptococcus mutans* biofilm colony forming units (CFU/ml) formation across all three groups was found to be 0.16 ± 0.11 (Table 1). When examining the specific polishing methods, the Shofu Super Snap mini kit exhibited a mean CFU/ml value of 0.24 ± 0.07, the aluminum oxide paste demonstrated a lower mean of 0.03 ± 0.05, and the propol polishing paste showed a mean of 0.23 ± 0.05 (Table 2). These findings suggest that the choice of polishing material significantly influences the biofilm formation of *Streptococcus mutans*. The aluminum oxide paste demonstrated the lowest biofilm formation, indicating its potential effectiveness in reducing bacterial adhesion compared to the other materials.

**Table 1:** Descriptive statistics of the Streptococcus mutans biofilm colony-forming units per milliliter (CFU/ml) formation for all the three groups. CFU/ml: colony-forming units per milliliter; N: sample size; mean ± SD: mean ± standard deviation.

Study parameter	N	Mean ± SD
CFU/ml	30	0.16 ± 0.11

**Table 2:** Descriptive statistics of the Streptococcus mutans biofilm colony-forming units per milliliter (CFU/ml) formation for the groups. CFU/ml: colony-forming units per milliliter; mean ± SD: mean ± standard deviation.

Mutans against three different polishing material		CFU/ml
		Mean ± SD
Streptococcus mutans	Shofu Super Snap mini kit	0.24 ± 0.07
	Aluminum oxide paste	0.03 ± 0.05
	Propol polishing paste	0.23 ± 0.05

For the surface roughness, the mean Ra values were 0.75 ± 0.21, 1.30 ± 0.25, and 2.04 ± 0.75 for the Shofu Super Snap mini kit, aluminum oxide paste, and propol polishing paste, respectively. The mean root mean square roughness (Rq) values were 1.07 ± 0.28, 1.77 ± 0.30, and 2.40 ± 0.78 for the Shofu Super Snap mini kit, aluminum oxide paste, and propol polishing paste, respectively. The mean average maximum height (Rz) values were 3.99 ± 1.06, 8.17 ± 0.59, and 8.98 ± 2.90 for the Shofu Super Snap mini kit, aluminum oxide paste, and propol polishing paste, respectively (Table 3). These results infer that the Shofu Super Snap mini kit has the roughest surface among the three materials, followed by propol polishing paste and aluminum oxide paste.

**Table 3:** Descriptive statistics for the surface roughness with respect to the groups. Ra: roughness average; Rq: root mean square roughness; Rz: average maximum height; mean ± SD: mean ± standard deviation.

Group	Ra	Rq	Rz
	Mean ± SD	Mean ± SD	Mean ± SD
Shofu Super Snap mini kit	2.04 ± 0.75	2.40 ± 0.78	8.98 ± 2.90
Aluminum oxide paste	0.75 ± 0.21	1.07 ± 0.28	3.99 ± 1.06
Propol polishing paste	1.30 ± 0.25	1.77 ± 0.30	8.17 ± 0.59

Inferential statistics

On comparing the mean values of 0.24 ± 0.07, 0.03 ± 0.05, and 0.23 ± 0.05 for the Shofu Super Snap mini kit, aluminum oxide paste, and propol polishing paste, it was found that they were statistically very highly significant (p < 0.001) (Table 4).

**Table 4:** Comparison of the Streptococcus mutans biofilm colony-forming units per milliliter (CFU/ml) formation with respect to the three groups of polishing material. ^A ^Test used was the Kruskal-Wallis test. * P < 0.05 is statistically significant. ** P < 0.01 is statistically highly significant. *** P < 0.001 is statistically very highly significant. CFU/ml: colony-forming units per milliliter; mean ± SD: mean ± standard deviation.

Group		CFU/ml	
		Mean ± SD	
Shofu Super Snap mini kit		0.24 ± 0.07	
Aluminum oxide paste		0.03 ± 0.05	
Propol polishing paste		0.23 ± 0.05	
P-value^A^		<0.001***	

On comparing the surface roughness among the three groups in the Ra category, it was found to be statistically very highly significant (p < 0.001). This was similar in the Rq and Rz groups as well (p < 0.001) (Table 5).

**Table 5:** Comparison of the surface roughness with respect to the three groups of polishing material. ^A^ Test used was the Kruskal-Wallis test. * P < 0.05 is statistically significant. ** P < 0.01 is statistically highly significant. *** P < 0.001 is statistically very highly significant. Ra: roughness average; Rq: root mean square roughness; Rz: average maximum height; mean ± SD: mean ± standard deviation.

Group	Ra	Rq	Rz
	Mean ± SD	Mean ± SD	Mean ± SD
Shofu Super Snap mini kit	2.04 ± 0.75	2.40 ± 0.78	8.98 ± 2.90
Aluminum oxide paste	0.75 ± 0.21	1.07 ± 0.28	3.99 ± 1.06
Propol polishing paste	1.30 ± 0.25	1.77 ± 0.30	8.17 ± 0.59
P-value^A^	<0.001***	<0.001***	<0.001***

SEM analysis

**Figure 2:**
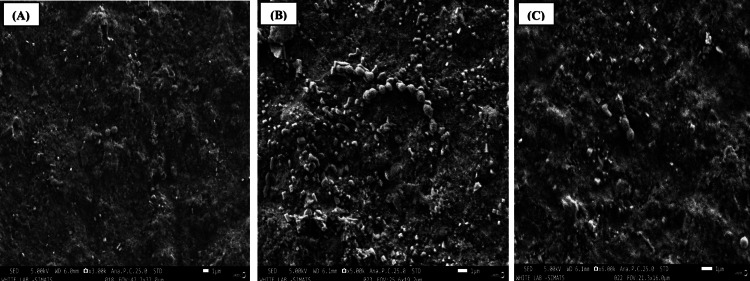
Scanning electron microscopy analysis of aluminum oxide polishing discs - Group 1. (A) 3x magnification; (B) 5x magnification; (C) 6x magnification.

**Figure 3:**
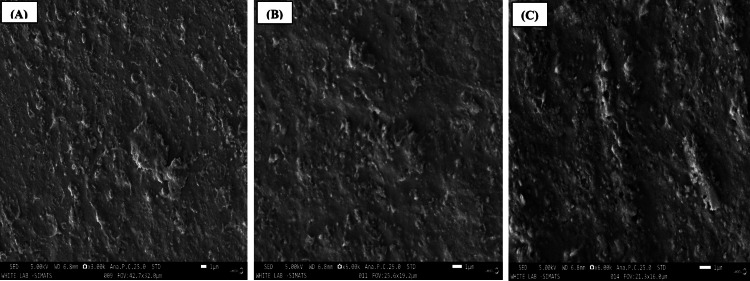
Scanning electron microscopy analysis of aluminum oxide paste - Group 2. (A) 3x magnification; (B) 5x magnification; (C) 6x magnification.

**Figure 4:**
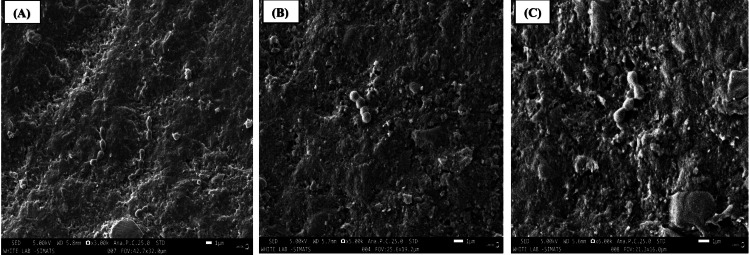
Scanning electron microscopy analysis of propol polishing paste - Group 3. (A) 3x magnification; (B) 5x magnification; (C) 6x magnification.

The SEM images are shown as follows: Figure 2 illustrates the SEM analysis of aluminum oxide polishing discs (Group 1), Figure 3 presents the SEM analysis of aluminum oxide paste (Group 2), and Figure 4 depicts the SEM analysis of propol polishing paste (Group 3). These SEM images are exemplary examples of the biofilm development of *S. mutans* on the specimen surfaces. Colonies and isolated *S. mutans* were detected in every group following 24-48 hours in vitro biofilm development period. In a descriptive analysis, the first and third specimen groups showed the greatest number of bacteria on their surface, with large adherent aggregates. The results indicate a significant interaction effect among the various polishing methods (p < 0.001). A positive association was observed between the values obtained for SEM, using the CFU/ml.

## Discussion

Temporization crown materials provide several benefits for the rehabilitation of primary teeth. Firstly, they protect the prepared tooth structure, shielding it from further decay and mechanical damage, which is crucial for maintaining the integrity and longevity of primary teeth [5]. Additionally, temporization crowns maintain occlusal function by providing a stable chewing surface, preventing occlusal disharmony and potential malocclusion. Aesthetically, these crowns enhance the appearance of primary teeth, helping boost the child’s confidence and social interactions [13,14]. They also prevent sensitivity by covering the exposed tooth and protecting it from thermal, chemical, and mechanical stimuli. However, research indicates that the surfaces of temporization materials are prone to bacterial adhesion, a key factor in the formation of biofilms in the marginal areas of restorations [15]. Cariogenic biofilm formation is a significant etiological factor in secondary caries, a primary cause of failure in temporization crowns.

This in vitro study aimed to evaluate and compare biofilm formation on temporization crown materials used in the rehabilitation of primary teeth, when polished with different materials, and to correlate these findings with surface roughness. The results revealed significant differences in biofilm formation and surface roughness depending on the polishing material. Aluminum oxide paste was the most effective, producing the smoothest surface with the lowest biofilm formation. Conversely, the Shofu Super Snap mini kit, though commonly used, resulted in the roughest surface and the highest accumulation of biofilm.

While numerous studies have investigated polishing materials, including comparisons with diamond polishing paste, no previous research has directly compared aluminum oxide paste, propol polishing paste, and the Shofu Super Snap mini kit. To the best of our knowledge, this study is the first to evaluate these specific materials in relation to surface roughness and biofilm formation. This unique comparison provides novel insights into the effectiveness of different polishing techniques on temporization materials, highlighting the significance of material choice in minimizing bacterial adhesion and biofilm growth.

The accumulation of biofilm on provisional restorations is directly related to the roughness of their surfaces [9]. To minimize bacterial adherence, reduce the risk of caries, and prevent discoloration, these materials should be polished before cementation [3]. In this study, surface roughness was significantly influenced by the polishing techniques used. The Shofu Super Snap mini kit group exhibited the highest mean Ra value (2.04 μm), indicating the roughest surface among the three materials tested. The propol polishing paste group showed a slightly lower mean Ra value (1.30 μm), while the aluminum oxide paste group had the least surface roughness, with a mean Ra value of 0.75 μm. Kavut et al. also conducted a study comparing different polishing materials, reporting mean surface roughness values of 0.45 µm for the aluminum oxide abrasive discs, 0.27 µm for the light curing surface-sealing material group, 0.29 µm for the diamond polishing paste group, and 0.25 µm for the self-adhesive acrylic resin coat group [16]. In this study, the results of the Ra values ranged between 0.75 and 2.04 μm. These values meet the criterion that an acceptable polishing system should deliver a surface roughness of less than 10 μm. The results of this study are similar to those obtained by Borchers et al. [6].

Increased surface roughness, exposure of filler particles, and reductions in microhardness after in vitro exposure to biofilms have been associated with the degradation of resin composite surfaces. Schmalz proposed that cariogenic bacteria, particularly *Streptococcus mutans* and *Lactobacilli*, predominantly localize along the margins of composite fillings [17]. The effect of temporization material on biofilm growth is influenced by factors such as filler particle properties and the chemical composition of the biofilm matrix. It is well established that biofilm formation occurs more frequently on temporization surfaces than on sound enamel [3].

Buergers et al. and Montanaro et al. conducted in vitro studies that quantified bacterial adhesion to various materials, using *Streptococcus mutans* commercial strains to inoculate provisional restoration materials like bis-acrylic resins, polymethyl methacrylate, and direct restorative materials [18,19]. These studies used techniques to measure surface roughness and SEM to observe bacterial colonization [20]. Biofilms form when microorganisms such as bacteria, algae, and fungi congregate on biological and non-biological surfaces [21]. Microorganisms typically require a humid environment to develop biofilms, as they cannot withstand dehydration [21]. Biofilm formation serves as a defense against external stress in certain environments [22], and life activities within biofilms differ significantly from those in the planktonic state [21]. Factors such as an aqueous environment, acidic fluid intake, and temperature fluctuations also contribute to surface degradation [23,24]. Mazurek-Popczyk et al. reported that raw materials produce the highest levels of biofilm, polished resins produce less, and glazed surfaces produce the least [15]. Bacterial adherence is stronger on hydrophilic surfaces than on hydrophobic ones, and the topography and roughness of a material's surface significantly influence bacterial adherence [25,26].

In this study, *Streptococcus mutans* was used as the cariogenic bacterium, and bacterial staining techniques were employed to confirm biofilm formation on temporization materials. Bacterial staining allowed for both qualitative and quantitative assessment of biofilm production by evaluating CFUs and using SEM [27]. The entire surface of each specimen was assessed, yielding comprehensive results.

The mean CFU values for the first, second, and third groups were 0.24 CFU/ml, 0.03 CFU/ml, and 0.23 CFU/ml, respectively. The first group exhibited the highest CFU value (p < 0.001), with a mean of 0.24 CFU/ml. Statistically significant differences in mean CFU values were observed between the Shofu Super Snap mini kit, aluminum oxide paste, and propol polishing paste groups (p < 0.001). SEM analysis revealed colonies and isolated *Streptococcus mutans* in all groups after 24-48 hours of in vitro biofilm development. The first and third specimen groups showed the highest bacterial counts, characterized by large adherent aggregates, demonstrating a positive correlation between SEM observations and CFU measurements.

This study also revealed that the lowest surface roughness and CFU counts were observed in the group treated with aluminum oxide paste, supporting the correlation between smoother surfaces and reduced biofilm formation on temporization materials.

Our study tested hypotheses regarding the effects of different polishing materials on biofilm formation on temporization materials. The null hypothesis, stating that there would be no change in biofilm formation with variations in polishing materials, was supported in some aspects. However, the alternate hypothesis, positing that different polishing materials could influence biofilm formation, was significantly supported in other areas. These findings highlight the importance of selecting appropriate polishing materials in dental practice to mitigate biofilm-related issues on temporization materials.

One limitation of this study is the use of a single bacterial species for biofilm measurement, which may not fully reflect the complex microbial environment of the oral cavity. While a single-species biofilm model provides controlled conditions and reproducible results, it limits the ecological validity of the findings. To confirm the clinical relevance of our findings, future studies should include in vivo evaluations. Clinical trials can help validate how these polishing materials perform under real-world conditions, assessing their impact on biofilm formation, material longevity, and patient outcomes.

## Conclusions

The surface characteristics of temporization crown material vary significantly depending on the polishing method used. In our study, the second group treated with aluminum oxide paste exhibited significantly lower biofilm development compared to both the first and third groups. This observation underscores the abrasiveness of aluminum oxide, which likely resulted in smoother specimen surfaces less conducive to biofilm adhesion and growth. The polishing methods evaluated in the study had varying effects on the production of biofilms, with the temporization material polished with aluminum oxide discs exhibiting the greatest biofilm development. These findings highlight the importance of selecting an appropriate polishing method to minimize biofilm formation and optimize surface smoothness.
